# Torsade de Pointes and Persistent QTc Prolongation after Intravenous Amiodarone

**DOI:** 10.1155/2012/673019

**Published:** 2012-03-05

**Authors:** Anna P. Kotsia, Georgios Dimitriadis, Giannis G. Baltogiannis, Theofilos M. Kolettis

**Affiliations:** ^1^Department of Cardiology, University Hospital of Ioannina, 1 Stavrou Niarxou Avenue, 45110 Ioannina, Greece; ^2^Department of Cardiology, Preveza General Hospital, 48100 Preveza, Greece

## Abstract

We report a case of torsade de pointes after intravenous amiodarone and concurrent hypokalemia. Despite treatment cessation and correction of electrolyte abnormalities, excessive QTc prolongation was noted, which persisted for 14 days. This prolonged course for QTc normalization may be attributed to the high rate of amiodarone loading and concurrent electrolyte disturbances coupled with possible underlying individual variability in pharmacokinetics.

## 1. Introduction

Amiodarone is a safe antiarrhythmic agent, associated with low proarrhythmia rates. However, a few cases of torsade de pointes have been reported after intravenous amiodarone [1–4]. By definition, drug-induced QTc prolongation normalizes after drug discontinuation, but, in the case of amiodarone, this time the interval is not well defined. We describe a case of excessive QTc prolongation after intravenous amiodarone that persisted for 14 days after treatment cessation. To our knowledge, such long-lasting QTc prolongation after intravenous amiodarone has not been described previously.

## 2. Case Description

**Figure 1:**
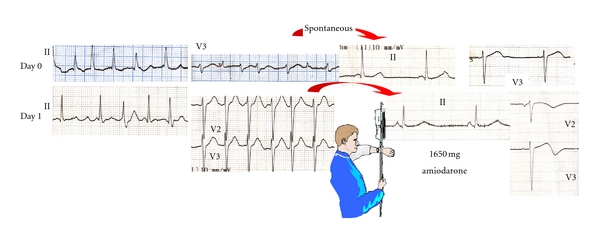
Baseline QTc. QTc interval was normal prior to amiodarone administration (day 0); after 1.65g intravenous amiodarone, QTc increased progressively.

A 57-year-old male, with unremarkable cardiovascular history, had a self-terminated episode of atrial fibrillation (Figure 1) one day prior to surgery for hiatus hernia. After the episode, the electrocardiogram was normal, with a QTc interval of 420 ms. Postoperatively, atrial fibrillation with rapid ventricular response was recorded, which was treated with intravenous amiodarone at an infusion rate of approximately 2 mg/min, following initial rapid administration of 150 mg over 10 minutes. Sinus rhythm was restored after 1 hour, but drug administration was continued for 12 hours, with a total amiodarone dosage of 1.65 g (Figure 1). 

**Figure 2:**
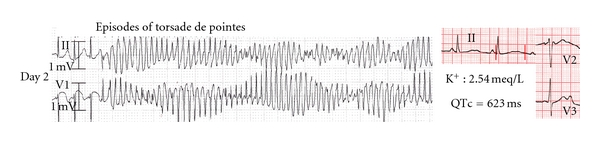
Torsade de pointes after amiodarone administration.

**Figure 3:**
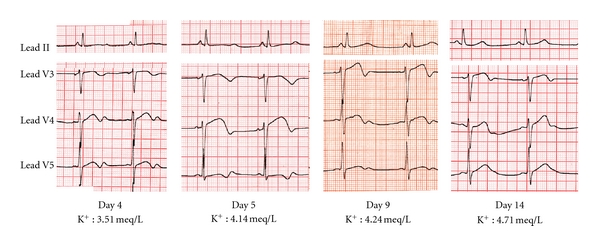
Time course of QTc prolongation. QTc prolongation persisted for 14 days after cessation of intravenous amiodarone. Note the (marginally) low serum K^+^ on the 4th hospital day.

QTc prolongation (at 623 ms) associated with hypokalemia (K^+^ : 2.54 meq/L) was noted and the patient was placed under continuous telemetry recording. Approximately 48 hours after amiodarone administration, torsade de pointes was recorded (Figure 2) and prompt defibrillation restored sinus rhythm. An acute coronary syndrome was ruled out and echocardiography was normal. Despite intravenous potassium and magnesium administration, two further episodes of torsade de pointes occurred, requiring the addition of isoproterenol infusion for 24 hours. At this time point, normal serum electrolyte values were recorded (Na^+^ : 138 meq/L, K^+^ : 4.79 meq/L, Mg^++^ : 1.7 meq/L) and the patient subsequently remained arrhythmia-free on telemetry recording. However, prolonged hospitalization was required, because QTc prolongation persisted until the 14th hospital day (Figure 3).

**Figure 4:**
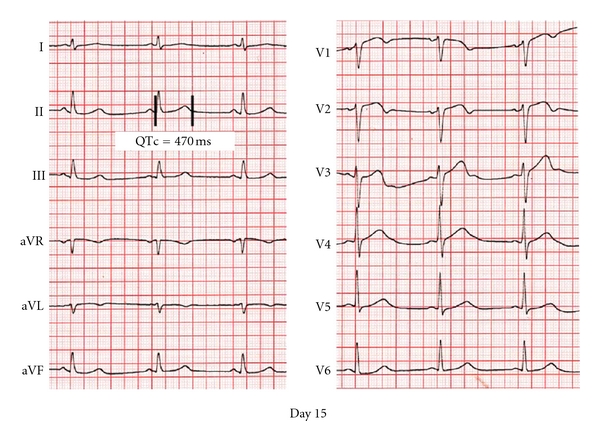
12-lead ECG at discharge. Prior to discharge, the QTc interval was 470 ms.

Blood biochemistry and thyroid function tests were normal and serum potassium was within normal limits during the remaining hospitalization period. Although Gitelman syndrome [5] was initially considered, this diagnosis was subsequently excluded, due to the absence of further electrolyte disturbances on repeated measurements during the follow-up period. The patient was discharged home on the 15th hospital day, at which point a QTc interval of 470 ms was recorded (Figure 4).

Andersen-Tawil Syndrome was excluded because there were no dysmorphic features and no history of muscle cramping or paralysis. The possibility of latent inherited long QT syndrome, unmasked by amiodarone, was considered according to previous experience [6], but the patient declined genetic testing. During a 10-month follow-up period, he remained asymptomatic and serial electrocardiograms were normal, as were electrocardiograms from first-degree relatives.

## 3. Discussion

Although side effects after intravenous amiodarone are rare and this therapy is generally considered safe, torsade de pointes has been previously described [1–4]. The causes of amiodarone-induced torsade de pointes are multifactorial, with electrolyte disturbances and concomitant medication with other QT-prolonging agents being the most common risk factors. In our patient, hypokalemia was considered a significant contributing factor to torsade de pointes. However, with the exception of low serum potassium (of borderline significance) documented on the 4th hospital day, long QT was noted despite normal potassium levels, thereby excluding hypokalemia alone as a cause of persistent QT prolongation.

The cause of hypokalemia in our patient is not entirely clear, but it should be most likely attributed to the combined effect of the (presumed) postoperative catecholamine release, along with nonrenal potassium loss, associated with the (short-term) use of nasogastric suction; in addition, isoproterenol infusion after the episodes of torsade de pointes may have been a contributory factor. This view is reinforced by the exclusion of other possible causes of hypokalemia, such as total body potassium depletion, including diuretic use and chronic alcoholism, as well as (primary and secondary) hyperaldosteronism.

Of note, the repolarization changes observed in precordial leads during the postoperative period may raise the possibility of Brugada syndrome, but this diagnosis was subsequently excluded, based on the absence of ST segment elevation in serial electrocardiograms during the hospitalization and follow-up period. It is likely that repolarization abnormalities (inverted T waves) in the precordial leads may have been caused by postoperative catecholamine surge, coupled with isoproterenol infusion; thus, acquired long QT syndrome due to stress-cardiomyopathy was considered, but the echocardiogram was not suggestive.

Given the very long half-life of amiodarone, electrocardiographic findings after oral administration may not resolve before several days or weeks after discontinuation of the drug [7]. However, despite some paucity of data, there is evidence from animal [8] and human [9] studies, in agreement with earlier reports [10], suggesting that pharmacokinetics after intravenous amiodarone display wide variation, but are generally more rapid than after oral administration. In concert with this data, the time course for QTc normalization after intravenous amiodarone in previous reports [2–4] ranged from 1 to 5 days.

Torsade de pointes has been previously reported [4] after 650 mg of intravenous amiodarone, followed by oral sotalol and ciprofloxacine; QTc normalized 3 days after discontinuation of these agents [4]. In another case, QTc normalized one day after 570 mg intravenous amiodarone [2]. However, in a further patient, QTc prolongation persisted for 5 days after 450 mg intravenous amiodarone, possibly due to concurrent methadone treatment [3]. In contrast with these findings, the most interesting observation in our case was the markedly prolonged time-course of QTc lengthening, which normalized 15 days after 1.65 g amiodarone administration.

Although the total amiodarone loading dose of 1.65 g in our case is higher than in previous reports [2–4], acute administration at dosages exceeding 2.0 g is common in clinical practice [11, 12]. However, despite the wide variability [12], the rate of amiodarone loading of 2 mg/min, chosen in the present paper, appears to be higher than most previously published regimens [13], rendering this a potential explanation for the observed side effect.

## 4. Conclusion

The time interval for QTc normalization after intravenous amiodarone varies, likely, due to the unique pharmacokinetics of this agent. Our patient developed acquired long QT syndrome in the setting of rapid amiodarone loading and hypokalemia, which resulted in torsade de pointes. In similar clinical scenarios, normalization of the QTc interval may take longer than previously reported and prolonged monitoring may be needed.
